# Dr. Hale's Reply to Mr. Brodie's Remarks on His Inaugural Dissertation

**Published:** 1816-04

**Authors:** E. Hale

**Affiliations:** Gardiner, Maine


*For the London Medical and Physical Journal.*

### Dr. Hale's *Reply to Mr. Brodie's Remarks on his Inaugural Dissertation.*

[From the New-England Journal of Medicine, &c.]

GENTLEMEN,

IN the New-England Journal for April last, are some remarks by Mr. Brodie upon my Inaugural Dissertation,* to which I beg leave to reply in your next number. I was absent on a journey immediately after receiving the number for April, until it was too late to write for your last.

Mr. Brodie objects to my experiments that they were not exact repetitions of his; and therefore not entitled to much consideration in estimating the causes of animal heat. It never could be doubted that experiments performed as his were, and by a gentleman of such distinguished character, were perfectly correct in all their details. Still it does not follow that similar results will be obtained from somewhat similar experiments, where some circumstances are different. Hence the propriety of such experiments being repeated by different persons. For, unless an exact imitation, in every particular, is studiously sought for, it can hardly be supposed that some circumstances will not differ, so as to lead to additional information. It may perhaps be an inaccuracy of language to call this a *repetition*, but, if it is, it is an inaccuracy that can never mislead, where the details of the experiments are particularly given.

Notwithstanding Mr. Brodie's remarks, therefore, I cannot but consider the circumstances in which my experiments differ from his, as the most important part of them. I will notice the several particulars mentioned by Mr. B. in the same order he has done.

1. Mr. Brodie used rabbits for his experiments, whereas I used dogs for some of mine, and cats for others. The former

* Copied from the Lon. Med. and Phys. Journal, vol. xxxii, p. 295.

former were, it seems, more readily obtained by him of the same age and size, and more easily managed; to me the latter offered the greatest facilities. Besides, he had already ascertained what would be the effect of artificial respiration in those animals. Had the result with different animals been the same as with those, it would have added strength to his conclusions against the chemical theory of animal heat. But that they were different, by no means disproves them. In my dissertation, I stated decidedly that I considered the state of the living principle to have a very powerful influence in producing heat, and gave some experiments performed with the express view of ascertaining, as far as possible, how far this influence extends. Artificial respiration evidently prolongs many of the vital, as well as the chemical actions. And it appears from Mr. Brodie's remarks, that the vitality is preserved much more completely in dogs and cats, than it is in rabbits. Here then is a very considerable reason for the difference of our results.

2. Mr. Brodie complains that I did not decapitate the animals in my experiments. But Mr. B. had already shown that the effect upon the respiration was the same, whether the spinal marrow merely was divided, or the head separated. So far as the chemical results were concerned, therefore, the former mode was equally certain, and more convenient. For, as the same degree of division was practised, on both animals in each experiment, the comparison between them was more perfect than if the head was separated. The effects of vitality in producing heat, had not much excited my attention until a subsequent part of the investigation.

3. I was not particular in examining the respired air. Mr. Brodie observes, "Had he found, as I did, that the animals in which the artificial respiration was employed, consumed as much oxygen, and evolved as much carbonic acid, as under ordinary circumstances, he would have regarded his experiments as equally conclusive with mine against the chemical theory of animal heat." I confess I cannot perceive upon what this opinion is founded. So far was I from considering the different circumstances of natural and artificial breathing, as an argument in favour of any theory, that I actually took it for granted the chemical effects were the same. They had been proved to be so by Mr. Brodie; and I found in one or two instances, when I examined, that carbonic acid was expelled, and that *always* the changes in the blood, which are usually attributed to chemical actions, were completely and brilliantly accomplished. I do not perceive the necessity, therefore, of examining very particularly what is so well established.

Mr.

Mr. Brodie seems to consider my experiments as professedly designed to support the chemical theory. In this he is mistaken. At the time I commenced experimenting, I had no decided opinion upon the subject: nor indeed when I had finished was I at all satisfied. I was very far from imagining that I had decided the question. That artificial respiration, under some circumstances, is the means of producing animal heat, and that this heat is at least in some degree owing to prolongation of the vital actions, is I think pretty clearly shown by my experiments. Whether any of this is entitled to the appellation of positive knowledge, I leave to others to decide.

I am, Gentlemen, your obedient Servant,

*Gardiner, Maine, Sept.* 15, 1815. E. HALE, Jun.

---

*For the London Medical and Physical Journal.*

